# Editorial: Information theory meets deep neural networks: theory and applications

**DOI:** 10.3389/fnins.2024.1448517

**Published:** 2024-07-15

**Authors:** Anguo Zhang, Qichun Zhang, Kai Zhao

**Affiliations:** ^1^Institute of Microelectronics, University of Macau, Taipa, China; ^2^School of Creative and Digital Industries, Buckinghamshire New University, Bradford, United Kingdom; ^3^School of Automation, Chongqing University, Chongqing, China

**Keywords:** artificial neural networks, information theory, information bottleneck, deep learning—artificial intelligence, deep neural networks (DNNs)

We are delighted to introduce this Research Topic, titled “*Information Theory Meets Deep Neural Networks: Theory and Applications*”. Deep neural networks (DNNs) have become a focal point in machine learning research, achieving impressive results across various tasks. However, understanding their workings and mechanisms remains challenging (Samek et al., 2021; Gawlikowski et al., 2023). Information theory, a mathematical framework for representing and analyzing information, has been widely applied to study the fundamental characteristics of data, such as structure and distribution. In the context of DNNs, information theory has been instrumental in explaining and optimizing their performance (Zhang and Li, 2019; Zhang et al., 2022, 2023). For instance, the information bottleneck theory has shed light on the abstract representations of neural networks, while entropy and mutual information have been used to evaluate model complexity and generalization performance (Wu et al., 2023). This Research Topic aims to explore the intersection of information theory and DNNs, two fields that have profoundly impacted the understanding and advancement of neural networks and their applications. The synergy between these disciplines offers promising avenues for developing more efficient, robust, and interpretable AI systems. In this Research Topic, we present four papers that illustrate the breadth and depth of research at this intersection, highlighting innovative methodologies and their applications in various domains.

You and Wang proposed a novel approach to genealogy layout recognition. Recognizing the significance of genealogies in cultural heritage, the authors introduced a sublinear information bottleneck (SIB) for feature extraction and a two-stage deep learning model combining SIB-ResNet and SIB-YOLOv5. This method surpassed existing state-of-the-art techniques, offering promising results in identifying and localizing components in genealogy images. This advancement not only aids in genealogy research but also in preserving cultural heritage through improved recognition technologies.

Li and Peng addressed the challenges of synthetic aperture radar (SAR) automatic target recognition (ATR). The study introduced a data augmentation technique that mitigates SAR image noise and a weighted ResNet with residual strain control. This approach not only enhances computational efficiency but also improves recognition accuracy, significantly reducing training time and data requirements. The experimental results demonstrated the superior performance of this method, paving the way for more efficient SAR ATR systems.

Alazeb et al. focused on shifting to the realm of robotic environments and scene classification. The paper presented a robust framework for multi-object detection and scene understanding, leveraging advanced visual sensor technologies and deep learning models. By integrating preprocessing, semantic segmentation, feature extraction, and object recognition, the proposed system achieved remarkable accuracy on standard datasets such as PASCALVOC-12, Cityscapes, and Caltech 101. This work represented a significant step forward in enhancing the capabilities of vision-based systems in various applications, from autonomous driving to augmented reality.

Finally, Chen et al. delved into the theoretical aspects of neural network training. The authors propose a novel method for adaptive learning rate estimation in restricted Boltzmann machines (RBMs) using rectified linear units (ReLUs). By providing mathematical expressions for adaptive learning step calculation, this approach optimized the learning rate dynamically, improving the generalization ability and reducing the loss function more effectively than traditional methods. This theoretical contribution offers valuable insights into the optimization of unsupervised learning algorithms.

In conclusion, this Research Topic showcased the innovative research at the crossroads of information theory and deep neural networks. The contributions presented here not only advance theoretical understanding but also demonstrate practical applications that hold the potential to transform various fields. We extended our gratitude to the authors for their exceptional work and to the reviewers for their rigorous evaluation. We hope this Research Topic inspires further research and collaboration in this exciting domain.

## Author contributions

AZ: Resources, Writing – original draft. QZ: Writing – review & editing. KZ: Writing – review & editing.
